# Elective Recurrent Inguinal Hernia Repair: Value of an Abdominal Wall Surgery Unit

**DOI:** 10.1007/s00268-023-07080-8

**Published:** 2023-06-02

**Authors:** V. Rodrigues-Gonçalves, M. Martínez-López, M. Verdaguer-Tremolosa, P. Martínez-López, M. López-Cano

**Affiliations:** General Surgery Department, Abdominal Wall Surgery Unit, Hospital Universitari Vall d´Hebron, Universitat Autònoma de Barcelona, Paseo Vall d`Hebron 119-129, 08035 Barcelona, Spain

## Abstract

**Background:**

The aim of this study was to analyze the impact of an abdominal wall surgery unit on postoperative complications (within 90 days postoperatively), hernia recurrence and chronic postoperative inguinal pain after elective recurrent inguinal hernia repair.

**Methods:**

We conducted a retrospective cohort study of all adult patients who underwent elective recurrent inguinal hernia repair between January 2010 and October 2021. Short- and long-term outcomes were compared between the group of patients operated on in the abdominal wall surgery unit and the group of patients operated on by other units not specialized in abdominal wall surgery. A logistic regression model was performed for hernia recurrence.

**Results:**

A total of 250 patients underwent elective surgery for recurrent inguinal hernia during the study period. The patients in the abdominal wall surgery group were younger (*P* ≤ 0.001) and had fewer comorbidities (*P* ≤ 0.001). There were no differences between the groups in terms of complications. The patients in the abdominal wall surgery group presented fewer recurrences (15% vs. 3%; *P* = 0.001). Surgery performed by the abdominal wall surgery unit was related to fewer recurrences in the multivariate analysis (HR = 0.123; 95% CI = 0.21–0.725; *P* = 0.021).

**Conclusions:**

Specialization in abdominal wall surgery seems to have a positive impact in terms of recurrence in recurrent inguinal hernia repair. The influence of comorbidities or type of surgery (i.e., outpatient surgery) require further study.

## Introduction

Inguinal hernia repair is one of the most frequent procedures performed by a general surgeon [1]. Despite being a common intervention, recurrence rates up to 15% have been reported [2]. The risk of hernia relapse in recurrent inguinal hernias is greater than the risk of relapse after repair of the primary hernia [3, 4]. For this reason, it has been suggested that operations in these patients be performed by experienced surgeons [5]. However, surgical specialization in abdominal wall surgery continues to be an area poorly studied in the literature, even more so in the case of recurrent inguinal hernia. Although clinical guidelines recommend treating these patients in specialized centers [6], little is known about the impact of specialization on short- and long-term postoperative outcomes in this difficult setting.

The objective of our study is to determine the impact of the surgeon’s specialization and experience in abdominal wall surgery on the postoperative complications (within 90 days postoperatively), hernia recurrence and chronic postoperative inguinal pain of elective recurrent inguinal hernia repair.

## Methods and patients

### Study design and setting

A retrospective cohort study was performed at Vall d´Hebron University Hospital between January 2010 and October 2021. All adult patients who underwent surgery for recurrent inguinal hernia were identified from a prospectively maintained database of our General Surgery Department. Patients with elective repair for recurrent inguinal hernia were selected for analysis, and the data were collected through a retrospective review of medical and surgical records. This study was conducted in accordance with the Declaration of Helsinki, and the Strengthening the Reporting of Observational Studies in Epidemiology (STROBE) [7] and Reporting of Studies Conducted Using Observational Routinely Collected Health Data (RECORD) [8] requirements for observational studies were applied.

Patients were classified according to the surgical unit that performed the surgical repair into patients operated on in the abdominal wall surgery unit (AWS group) and patients operated on by surgeons not specialized in abdominal wall surgery (NAWS group).

In our hospital, there are two surgery departments with completely independent management models, one for digestive surgery and another for hepatobiliary and pancreatic surgery and liver transplantation. The digestive surgery department is organized into specialized units with exclusive dedication to abdominal wall, colorectal, gastroesophageal, endocrine, and bariatric surgery. Due to the presence of two independent surgical departments, some repairs were performed by surgeons outside the abdominal wall surgery unit.

The abdominal wall surgery unit comprises three senior surgeons who specialize in abdominal wall surgery, two fellows, and one resident and performs approximately 500 inguinal hernia repairs per year, including majority of outpatient inguinal hernia surgeries at our hospital. A surgeon is defined as an abdominal wall specialist with the following criteria: high surgical volume and a minimum of 5 years of surgical dedication to abdominal wall surgery [9, 10]. Our abdominal wall surgery unit meets the required criteria to be a hernia center published by different organizations [11, 12]. The rest of the surgeries were performed by the NAWS group comprising six surgeons specializing in other areas.

### Patients

The inclusion criteria were age ≥ 18 years and elective repair of recurrent inguinal hernia.

The exclusion criteria were patients < 18 years, patients who underwent emergency recurrent inguinal hernia repair, and patients with recurrent inguinal hernia who did not undergo surgery.

Patients were followed up by their surgeons at regular intervals. A follow-up visit was routinely performed 4 weeks after hospital discharge. More face-to-face visits were scheduled before or after the routine visit depending on the presence of postoperative complications.

For the purpose of this study, telephone interviews were conducted at the time of the study to assess the presence of chronic postoperative pain (CIPIP) and recurrence.

### Preoperative variables

Patient demographic data (age, sex, and body mass index) as well as clinical variables, including American Society of Anesthesiologists (ASA) classification, the Charlson score [13], and the presence of comorbidities (chronic obstructive pulmonary disease (COPD), cardiovascular disease, diabetes, chronic nephropathy, anticoagulant therapy, neurocognitive disorders, and smoking status), were collected. Variables related to the hernia included the side of the hernia, content, and type of hernia according to the EHS classification [6]. Regarding the primary hernia repair, the type of approach (anterior or posterior) and the type of repair (tissue or mesh) were collected.

### Operative variables

The procedures documented in the operative records were reviewed to classify the repair approaches for recurrent hernias into anterior and posterior. An open transinguinal repair using mesh was considered an open anterior approach. A posterior approach involved open or laparoscopic posterior access to the preperitoneal space without entering the inguinal canal from the front and exposure of all myopectineal orifices to allow hernia repair with placement of a prosthesis.

Recurrent hernia repairs were classified according to whether they were based on guideline recommendations. Repairs based on clinical guidelines included cases in which the primary hernia had been operated on using an anterior tissue or mesh approach and the recurrence was repaired with a posterior approach (open preperitoneal or laparoscopic) and cases in which the primary hernia had been treated with a posterior approach and the recurrence was repaired with an anterior approach using mesh. The cases in which the primary hernia and recurrence were operated on using the same type of approach (anterior or posterior) were considered repairs not based on clinical guidelines.

The type of surgical approach was selected at the surgeon´s discretion. The type of anesthesia was determined by the anesthesiologist. Some patients underwent outpatient surgery according to the preoperative assessment by the anesthesiologist. Given that these patients had fewer comorbidities, a subgroup analysis was performed excluding these patients to create more homogeneous groups.

### Postoperative variables

The postoperative variables collected were complications (within 90 days postoperatively), CPIPs and hernia recurrence. Postoperative complications were defined as any condition that could influence the outcomes or prolong the length of hospital stay. The severity of postoperative complications was graded using the Clavien–Dindo classification [14].

Hernia recurrence was determined by review of operative notes reporting any reoperations for hernia recurrence, physical evaluation by the surgeon, or by telephone interview based on a patient-reported outcome measure questionnaire: the Ventral Hernia Recurrence Inventory (VHRI) [15]. The VHRI is a validated tool used to assess the presence of recurrence in both ventral [16] and inguinal hernias [15]. CPIP was defined as persistent pain lasting more than 3 months after surgery [17]. CPIP was assessed using the last question of the VHRI questionnaire: “Do you have pain or other physical symptoms at the site?” made in the telephone interview. If presenting any positive response in the VHRI, patients were strongly recommended to schedule a face-to-face visit for a physical evaluation. The last face-to-face postoperative visit was considered the last follow-up date in the patients who did not respond to follow-up telephone interviews.

### Statistical analysis

Continuous variables are reported as medians and interquartile ranges (IQRs) and were analyzed using Student´s t test or the Mann–Whitney U test as needed. Categorical variables are reported as counts and percentages and were compared using the chi-square test or Fisher´s exact test as needed. A logistic regression model was performed for hernia recurrence. The inclusion of variables in the model was based on their significance in the univariate analysis (*P* < 0.05) and on clinical consensus. Recurrence results are reported as hazard ratios with 95% confidence intervals. The Kaplan–Meier method was used to estimate the cumulative recurrence rate, and significance was tested with the log-rank test. *P* < 0.05 was accepted as a significant statistical value. For statistical analysis, SPSS (IBM SPSS Statistics 23) was used.

## Results

### Demographics

Of a total of 250 patients who underwent elective repair of recurrent inguinal hernia from January 2010 to October 2021, 214 (86%) had first-episode recurrence, and 36 (14%) had multiple recurrences of inguinal hernia. All repairs were unilateral recurrences. A total of 196 patients (78%) were operated on by surgeons from the abdominal wall surgery unit, and 54 (22%) were operated on in other general surgery units. Most patients with recurrence in the AWS group underwent outpatient surgery (*n* = 138; 67%), while only ten patients (10%) in the NAWS group underwent surgery in this setting. Patients in the NAWS group were older (*P* ≤ 0.001) and had more comorbidities (*P* ≤ 0.001) (Table 1).

**Table 1:** Patient characteristics of study population

Variables	Total (*n* = 250)	NAWS group (*n* = 54)	AWS group (*n* = 196)	*P* values
Age (yr)[median (IQR)]	66.5 (54 – 77)	75 (67 – 81)	63 (51 – 74)	< 0.001
Sex [n, (%)]				0.473
Male	225 (90)	50 (93)	175 (89)	
Female	25 (10)	4 (7)	21 (11)	
BMI (kg/m^2^) [median (IQR)]	25.4 (23.8 – 27.5)	24.5 (23.4 – 26.3)	25.7 (24—27.8)	0.031
ASA score				< 0.001
I/II [n, (%)]	180 (72)	24 (44)	156 (80)	
III/IV [n, (%)]	70 (28)	30 (56)	40 (20)	
Charlson score [median (IQR)]	3 (1 -5)	5 (4—7)	3 (1 – 5)	< 0.001
Comorbidity [n, (%)]	149 (60)	46 (85)	103 (53)	< 0.001
Cardiovascular disease [n, (%)]	86 (34)	28 (52)	58 (30)	0.002
Chronic obstructive pulmonary disease [n, (%)]	46 (18)	20 (37)	26 (13)	< 0.001
Chronic nephropathy [n, (%)]	18 (7)	8 (15)	10 (5)	0.031
Neurocognitive disorders [n, (%)]	17 (7)	10 (19)	7 (4)	0.001
Diabetes [n, (%)]	28 (11)	10 (19)	18 (9)	0.054
Active smoking [n, (%)]	36 (14)	10 (19)	26 (13)	0.330
Anticoagulant treatment [n, (%)]	30 (12)	17 (31)	13 (7)	< 0.001
Comorbidity more than one [n, (%)]	75 (30)	29 (54)	46 (23)	< 0.001
Hernia type [n, (%)]				0.305
Lateral	101 (41)	28 (52)	73 (37.5)	
Medial	87 (35)	14 (26)	73 (37.5)	
Femoral	20 (8)	4 (7)	16 (8)	
Combined	36 (14)	6 (11)	30 (15)	
Others	6 (2)	2 (4)	4 (2)	
Hernia side [n, (%)]				0.771
Right	134 (54)	28 (52)	106 (54)	
Left	116 (46)	26 (48)	90 (46)	
Inguinoescrotal hernia [n, (%)]	21 (8)	5 (9)	16 (8)	0.784
Multirecurrent hernia [n, (%)]	36 (14)	7 (13)	29 (15)	0.734
Hernia sac contents [n, (%)]				0.167
Omentum	22 (9)	2 (4)	20 (10)	
Small bowel	3 (1.2)	1 (2)	2 (1)	
Colon	16 (6)	1 (2)	15 (8)	
Bladder	8 (3.2)	1 (2)	7 (3.5)	
Other	1 (0.4)	0 (0)	1 (0.5)	
Not reported	192 (77)	49 (90)	143 (73)	
Empty	8 (3.2)	0 (0)	8 (4)	
Guideline-based repair [n,	185 (74)	8 (15)		< 0.001
(%)]			177 (90)	
Type of primary hernia repair approach [n, (%)]				0.005
Anterior	212 (85)	52 (96)	160 (82)	
Posterior	38 (15)	2 (4)	36 (18)	
Type of primary hernia repair [n, (%)]				< 0.001
Tissue repair	100 (40)	35 (65)	65 (33)	
Mesh repair	150 (60)	19 (35)	131 (67)	
Emergency presentation of primary hernia [n, (%)]	15 (6)	5 (9)	10 (5)	0.327
Type of anesthesia [n, (%)]				< 0.001
Spinal	143 (57)	38 (70)	105 (54)	
Local alone	4 (2)	0 (0)	4 (2)	
General	103 (41)	16 (30)	87 (44)	
Type of hernia repair approach				< 0.001
[n (%)]				
Anterior	68 (27)	47 (87)	21 (11)	
Posterior	182 (73)	7 (13)	175 (89)	
Type of procedure [n (%)]				< 0.001
Lichtenstein	56 (22)	36 (67)	20 (10)	
Other anterior technique	12 (5)	11 (20)	1 (0.5)	
Open preperitoneal mesh	160 (64)	6 (11)	154 (78.5)	
Laparoscopic	22 (9)	1 (2)	21 (11)	
Postoperative complication [n (%)]	53 (21)	14 (26)	39 (20)	0.337
Clavien–Dindo classification of postoperative complications [n, (%)]				0.050
None	197 (79)	40 (74)	157 (80)	
I	48 (19)	11 (20)	37 (19)	
II	3 (1)	1 (2)	2 (1)	
III A	1 (0.5)	1 (2)	0 (0)	
III B	1 (0.5)	1 (2)	0 (0)	
Wound hematoma [n, (%)]	29 (12)	12 (22)	17 (9)	0.006
Wound infection [n, (%)]	3 (1)	2 (4)	1 (0.5)	0.119
Wound seroma [n, (%)]	16 (6)	3 (6)	13 (7)	1.000
Ischemic orchitis [n, (%)]	2 (1)	2 (4)	0 (0)	0.046
Hernia Re-recurrence [n, (%)]	13 (5)	8 (15)	5 (3)	0.001
Chronic postoperative inguinal pain [n, (%)]	34 (14)	4 (7)	30 (15)	0.519

NAWS: not specialized in abdominal wall surgery

AWS: abdominal wall surgery

### Hernia characteristics

Comparing the characteristics of the primary hernia, abdominal wall unit surgeons had more recurrences after posterior repairs (open preperitoneal and laparoscopic) (*P* = 0.005) and after a previous mesh repair (*P* ≤ 0.001). Regarding the repair of recurrent hernia, abdominal wall surgeons performed a greater number of repairs based on clinical guidelines (*P* ≤ 0.001) and more frequently performed repairs by the preperitoneal approach (*P* ≤ 0.001), either open or laparoscopic (*P* ≤ 0.001) (Table 1).

### Postoperative outcomes

The overall rate of 90-day postoperative complications was 20% (*n* = 51). The most common postoperative complications included hematoma (*n* = 29; 12%), seroma (*n* = 16; 6%) and wound infection (*n* = 3; 1%). Patients in the NAWS group had a higher rate of wound hematoma (*P* = 0.006) and ischemic orchitis (*P* = 0.046). There were no significant differences between the groups in terms of seroma formation (*P* = 1) or wound infection (*P* = 0.119).

### Hernia recurrence

**Fig. 1:**
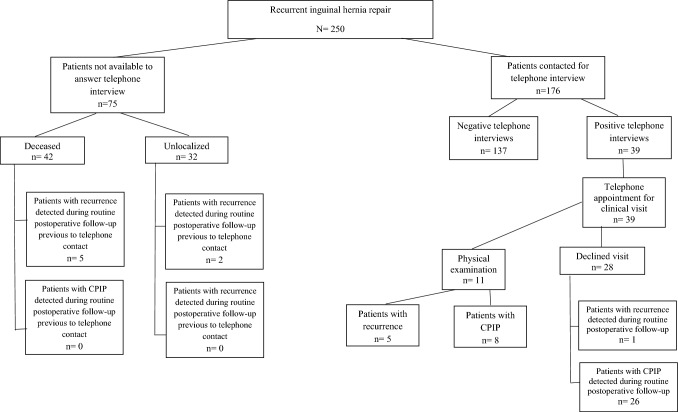
Flowchart of study cohort and long-term outcomes

The median follow-up was 58 months (IQR: 14.75–97). The flowchart of the patients included in the study with the long-term results is shown in Fig. 1. The recurrence rate of the entire series was 5% (*n* = 13). Patients operated on by surgeons not specialized in abdominal wall surgery had a significantly higher rate of recurrence than those operated on by abdominal wall surgeons (NAWS 15%vs. AWS 3%; *P* = 0.001).

Only procedures performed by the AWS unit were associated with fewer recurrences in multivariate analysis (HR = 0.123; 95% CI = 0.21–0.725; *P* = 0.021) (Table 2).

**Table 2:** Univariable and multivariable analysis of recurrence

	Recurrence			
Variables	Univariable analysis		Multivariable analysis	
	HR (95% CI)	P value	HR (95% CI)	P value
Patient age (y)		0.008		0.046
< 75 (*n* = 164)	1		1	
≥ 75 (*n* = 74)	0.206 (0.64—0.662)		0.287 (0.084 – 0.978)	
Sex		0.581		
Male (*n* = 214)	1			
Female (*n* = 24)	1.784 (0.229 – 13.926)			
BMI		0.647		
< 30 (*n* = 214)	1			
≥ 30 (*n* = 24)	1.612 (0.209 – 12–412)			
ASA score		0.219		
I/II (*n* = 168)	1			
III/IV (*n* = 70)	0.492 (0.159 – 1.523)			
Charlson score		0.139		
< 3 (*n* = 93)	1			
≥ 3 (*n* = 145)	0.375 (0.102 – 1.373)			
Comorbidity		0.330		
Yes (n = 144)	0.552 (0.167 – 1.834)			
No (*n* = 94)	1			
Cardiovascular disease		0.175		
Yes (*n* = 83)	0.463 (0.152 – 1.409)			
No (*n* = 155)	1			
Chronic Obstructive Pulmonary disease		0.214		
Yes (n = 45)	0.471 (0.144 – 1.543)			
No (n = 193)	1			
Chronic nephropathy		0.590		
Yes (*n* = 17)	21.872 (0.000 – 1,612,659.634)			
No (*n* = 221)	1			
Diabetes		0.618		
Yes (*n* = 28)	1.681 (0.218 – 12.939)			
No (*n* = 210)	1			
Active smocking		0.361		
Yes (*n* = 35)	26.258 (0.024 – 29,003.901)			
No (*n* = 203)	1			
Anticoagulant treatment		0.463		
Yes (*n* = 30)	0.463 (0.122 – 2.611)			
No (*n* = 208)	1			
Comorbidity more than one		0.954		
Yes (*n* = 74)	1.040 (0.281 – 3.842)			
No (*n* = 164)	1			
Femoral hernia		0.738		
Yes (*n* = 18)	1.422 (0.180 – 11.223)			
No (*n* = 220)	1			
Inguinoescrotal hernia		0.905		
Yes (*n* = 20)	0.883 (0.113 – 6.900)			
No (*n* = 218)	1			
Multirecurrent hernia		0.329		
Yes (*n* = 35)	26.535 (0.037 – 19,118.891)			
No (*n* = 203)	1			
Guideline-based repair		0.184		
Yes (*n* = 176)	2.142 (0.696 – 6.587)			
No (*n* = 62)	1			
Type of surgeon		0.007		0.021
NAWS (n = 54)	1		1	
AWS (*n* = 184)	0.140 (0.045 – 0.437)		0.123 (0.021 – 0.725)	
Primary hernia repair approach		0.822		
Anterior (*n* = 207)	1			
Posterior (*n* = 31)	0.841 (0.186 – 3.810)			
Primary hernia repair		0.145		
Tissue repair (*n* = 113)	0.414 (0.127 – 1.354)			
Mesh repair (*n* = 125)	1			
Recurrent hernia repair approach		0.028		0.521
Anterior (*n* = 67)	1		1	
Posterior (*n* = 171)	3.414 (1.146 – 10.176)		0.554 (0.091 – 3.367)	
Postoperative complication		0.294		
Yes (*n* = 48)	0.532 (0.163 – 1.730)			
No (*n* = 190)	1			

NAWS: not specialized in abdominal wall surgery

AWS: abdominal wall surgery

**Fig. 2:**
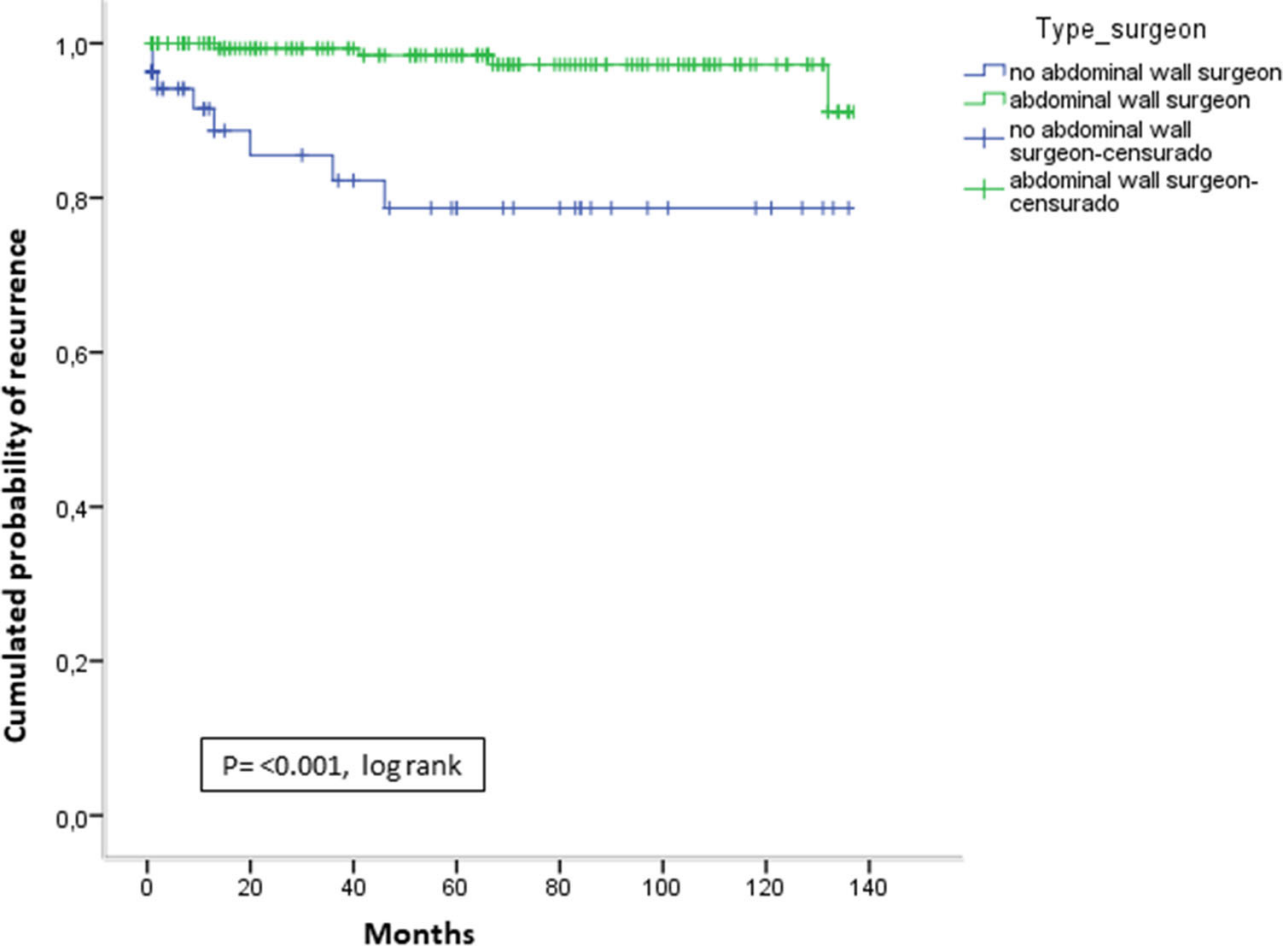
Cumulative probability of recurrence

In the cumulative recurrence curves, significant differences were observed in favor of the AWS group compared with the NAWS group (*P* ≤ 0.001, log rank) (Fig. 2).

### Chronic postoperative inguinal pain

The rate of CPIP in the entire series was 14% (*n* = 34). There were no significant differences between the patients operated on by general surgeons and those operated on by surgeons from the abdominal wall surgery unit (*P* = 0.519).

### Subgroup analysis

Patients were compared according to the type of surgeon, excluding those who underwent outpatient surgery. There were no significant differences between the groups in terms of age (*P* = 0.922) or ASA classification (*P* = 0.322). Regarding the presence of comorbidities, patients in NAWS group presented a higher percentage of COPD (*P* = 0.027) compared to patients in the AWS group. There were no significant differences between the groups in terms of the presence of chronic nephropathy (*P* = 0.546), active smoking (*P* = 0.982), and neurocognitive disorders (*P* = 0.089). Patients operated on by abdominal wall surgeons had more interventions after mesh repair (*P* ≤ 0.001) and a higher number of guideline-based repairs (*P* ≤ 0.001). Of note, a higher number of recurrences was observed in the group of patients operated on by surgeons from other surgery units (*P* = 0.001).

## Discussion

The present study shows that the specialization of the surgeon in abdominal wall surgery is one of the main factors in reducing recurrence after repair of recurrent inguinal hernias.

The role of specialization in general and digestive surgery has been previously evaluated in several areas, such as colorectal [18] and bariatric surgery [19]. These studies have shown better results in patients operated on in high-volume centers and by specialized surgeons [20]. In abdominal wall surgery, lower recurrence rates have been reported in incisional hernia repairs in patients operated on by specialized surgeons [12]. Regarding inguinal hernia surgery, previous studies have shown a reduction in recurrence rates with high-volume surgeons in primary hernia repairs [21]. In addition, better outcomes have been reported at specialized inguinal hernia repair centers compared with general hospitals in Canada [22]. In recurrent inguinal hernia, although clinical guidelines recommend management by expert surgeons [6], this aspect has been poorly studied in the literature.

Recurrent inguinal hernia repair still represents an important challenge for general surgeons as it is a technically demanding procedure and is associated with a high rate of postoperative morbidity and repeated recurrence [3]. The present study shows that the main factor in reducing recurrences in the elective repair of recurrent inguinal hernia is the surgeon´s specialization in abdominal wall surgery. A previous study showed a lower risk of chronic pain in patients operated on for recurrent inguinal hernia by high-volume surgeons [5]. Some authors have reported that the incidence of recurrence appears to depend to a great extent on the skill of the surgeon in both open and laparoscopic approaches [23]. However, the criteria for defining the proficiency of the hernia surgeon in most of these studies were based on annual case volume with arbitrary cutoff points. In our study, patients were operated on by surgeons from a unit specialized in abdominal wall surgery that met the proposed requirements to certify hernia centers in other European countries[9, 10], and significantly better results were obtained in terms of recurrence compared with surgeons from other specialties. The high volume and systematic repetition of techniques in a surgical protocol can improve and refine the skills of surgeons involved in a specific abdominal wall surgery unit.

The group of patients operated on by surgeons not specialized in abdominal wall surgery were older and presented more comorbidities, including COPD, which is a recognized risk factor for recurrence [6] that could have influenced the results observed in this group. However, in multivariate analysis, COPD was not a risk factor for recurrence in our study. This differences between the groups can be explained by the fact that in our surgery department, younger patients with fewer comorbidities underwent surgery on an outpatient basis, and the abdominal wall surgery unit is responsible for performing most inguinal hernia repairs in this regimen. Interestingly, when both groups were compared excluding patients who underwent outpatient surgery, no differences were observed in terms of age and most comorbidities; however, the differences in postoperative results were maintained in terms of lower recurrence rates in the AWS group.

Regarding the characteristics of the hernia, a higher percentage of patients with recurrences after a previous posterior approach and mesh techniques were operated on in the group of surgeons from the abdominal wall unit. Although these recurrences can be considered more difficult procedures, the rate of complications between the groups was similar, and even in the AWS group there was a lower rate of recurrence. This can also be explained by the fact that surgeons from the abdominal wall unit performed a higher number of guideline-based repairs (90% vs. 15%) than surgeons from other surgical units. In the literature, compliance with guideline-based repairs of 38.5% was reported, and nonguideline-based repairs were associated with higher rates of intraoperative complications, seroma formation, and recurrences [24]. Although there was greater adherence to the guidelines in the AWS group, compliance was not complete. This is demonstrated in the imbalance observed between patients originally operated on via a posterior approach (18%), in whom only 11% underwent an anterior approach. This is because the choice of technique ultimately depended on the surgeon´s criteria and patient-specific factors. In our study, there was a high percentage of patients who underwent regional anesthesia that would not be in accordance with guideline recommendations [6]. This can be explained by the fact that the decision on the type of anesthesia was at the discretion of the anesthesiologist. However, in the AWS group there was a significantly greater use of general anesthesia.

Despite the fact that preperitoneal approaches, both open and laparoscopic, have better results and offer clear benefits in this setting[25], in our series surgeons not specialized in abdominal wall surgery used conventional anterior approaches more frequently to repair recurrences. The preperitoneal approach avoids scarred and distorted tissues that can increase the risk of local complications, ischemic orchitis, and CPIP [26]. On the other hand, this approach allows complete exposure of the myopectineal orifice and allows the placement of a large mesh, potentially reducing the risk of a new recurrence. In our opinion, this reinforces the importance of specialized abdominal wall surgery units where adherence to surgical guidelines and protocols may be more feasible.

The incidence of CPIP in the present study was 14% without significant differences between the groups, while the incidence reported in the literature was as high as 27.6% depending on the type of approach, the definition of CPIP, and the measurement methods [23, 27, 28]. Although without significant differences, the percentage of patients with CPIP was higher than in the AWS group; this could be explained by the fact that in this group there were a greater number of multirecurrent hernias and recurrences after mesh repairs, factors that increase the risk of CPIP after recurrent inguinal hernia repairs [5]. The fact that no differences were found between the groups according to the surgeon´s specialization must be interpreted with caution for several reasons. First, due to the retrospective design of the study, it was not possible to obtain inguinal pain data prior to recurrence repair. Second, the indication to perform recurrent inguinal hernia repair is often determined by the degree of pain and disability, which may have caused a selection bias.

The current study has some limitations: (1) this is a single-center retrospective study that, due to its nature, could not collect some important variables, such as the classification of the primary hernia; (2) there are no clear criteria to define both a surgeon and an abdominal wall surgery unit; however, our unit complies with the requirements proposed to certify hernia centers in other European countries [9, 10]; (3) the surgical approach was performed at the surgeon´s discretion, which may lead to selection bias; (4) not all patients were personally examined as the follow-up was based on a telephone questionnaire, which could affect the number of total recurrences detected; however, the VHRI method is considered an adequate instrument to minimize the risk of missing a recurrent hernia [15]; (5) as not all patients could be contacted for telephone follow-up, reported rates of recurrence and CPIP could potentially underestimate the current rate; and 6) the fact that the AWS group had fewer comorbid conditions (i.e., COPD) may have influenced the results in terms of recurrence, however, in the subgroup analysis the results in favor of the group of patients operated on by abdominal wall surgery specialist remained when excluding those patients operated on in outpatient surgery.

In summary, in this specific type of hernia, the choice of the best surgical approach should be guided by experience, knowledge and implementation of protocols and clinical guidelines with a tailored approach allowing a low risk of surgical complications and fewer repeated recurrent inguinal hernias. The results of our study with a lower repeated recurrence rate support the idea that the elective repair of recurrent inguinal hernia should be performed in the context of a specialized abdominal wall surgery unit.
